# Field effects and ictal synchronization: insights from in homine observations

**DOI:** 10.3389/fnhum.2013.00828

**Published:** 2013-12-05

**Authors:** Shennan A. Weiss, Guy McKhann Jr, Robert Goodman, Ronald G. Emerson, Andrew Trevelyan, Marom Bikson, Catherine A. Schevon

**Affiliations:** Department of Neurology, Schevon Lab, Columbia UniversityNew York, NY, USA; Department of Neurosurgery, Columbia UniversityNew York, NY, USA; Department of Neuroscience, Newcastle UniversityNewcastle, UK; Biomedical Engineering, The City College of The City University of New YorkNew York, NY, USA

**Keywords:** ephaptic conduction, field effect, seizures, epilepsy, synchrony

## Abstract

It has been well established in animal models that electrical fields generated during inter-ictal and ictal discharges are strong enough in intensity to influence action potential firing threshold and synchronization. We discuss recently published data from microelectrode array recordings of human neocortical seizures and speculate about the possible role of field effects in neuronal synchronization. We have identified two distinct seizure territories that cannot be easily distinguished by traditional EEG analysis. The ictal core exhibits synchronized neuronal burst firing, while the surrounding ictal penumbra exhibits asynchronous and relatively sparse neuronal activity. In the ictal core large amplitude rhythmic ictal discharges produce large electric fields that correspond with highly synchronous neuronal firing. In the penumbra rhythmic ictal discharges are smaller in amplitude, but large enough to influence spike timing, yet neuronal synchrony is not observed. These *in homine* observations are in accord with decades of animal studies supporting a role of field effects in neuronal synchronization during seizures, yet also highlight how field effects may be negated in the presence of strong synaptic inhibition in the penumbra.

An electrical field effect occurs when currents associated with an extracellular field cross the cell membrane. If the current is significant the transmembrane potential (*V_m_* = *V*_intracelullar_ − *V*_extracellular_) will differ from the intracellular potential. If the transmembrane potential surpasses threshold it may initiate firing, or at reduced transmembrane polarization influence action potential timing (Radman et al., 2007a; Anastassiou et al., 2011), synaptic efficacy (Bikson et al., 2004), or other membrane processes (Faber and Korn, 1989; Jefferys, 1995; Weiss and Faber, 2010).

Field effects are thought to play a role in seizure initiation and propagation (Jefferys, 1995; Dudek et al., 1998). In the absence of synaptic transmission, non-synaptic mechanisms are sufficient to initiate and propagate seizure like activity in hippocampal slice models (Jefferys and Haas, 1982; Taylor and Dudek, 1982, 1984; Jiruska et al., 2010). Also, paired extra- and intracellular recordings of spontaneous paroxysmal events in cat neocortex *in vivo*, with synaptic transmission unaffected, have confirmed that fields associated with ictal discharges depolarize the neuronal membrane and can elicit action potentials (Grenier et al., 2003a,b).

These discharges are thought to be generated by large paroxysmal depolarizing shifts (Goldensohn and Purpura, 1963; Grenier et al., 2003a,b) mediated by glutamatergic synaptic transmission, high-voltage calcium spikes, and a persistent voltage-gated sodium current (Traub et al., 1993). The electric field associated with these currents ranges between 3–9 mV/mm (Pockberger et al., 1984; Jefferys, 1995). Early modeling studies found that the electric fields associated with ictal discharges can synchronize action potentials on a time scale of 1 ms (Traub et al., 1985). Moreover, in hippocampal slices neuronal synchrony during ictal discharges is modulated by changes in osmolality that can strengthen or weaken field effects (Bikson et al., 2003).

The mechanism by which field effects contribute to neural synchronization has been a subject of intense study. It is estimated that DC uniform fields alter the transmembrane potential in individual neurons at the soma (Radman et al., 2009) by 0.18 mV per mV/mm field strength (Deans et al., 2007). However, in hippocampal slices bathed in high K+ to elicit epileptiform activity exogenously pulsed uniform fields as small as 295 µV/mm could entrain neuronal firing (Francis et al., 2003). An explanation for the sensitivity of spike timing to weak electric fields may be that network interactions amplify small field effects experienced by all neurons across an extended territory (Parra and Bikson, 2004; Reato et al., 2010; Weiss and Faber, 2010) by modifying the spike timing of a significant portion of the population (Radman et al., 2007b; Anastassiou et al., 2011) and increasing the synchrony of chemical synaptic transmission in an auto-regenerative manner. Recordings from cortical neuronal ensembles, *in vitro* (Anastassiou et al., 2011), and *in vivo* (Ozen et al., 2010) confirm that population level spike coherence to exogenous non-uniform oscillating fields occurs at strengths ranging from 1–4 mV/mm.

If weak electric fields contribute to neuronal synchronization, it would be expected that neuronal synchrony would be observed during the large electric fields generated by ictal discharges in humans. Despite the importance of neural synchrony in seizures, there is a dearth of multi-electrode recordings demonstrating such synchrony over extended cortical territories. Recent recordings of partial seizures from the human cortex with the Utah microelectrode array (House et al., 2006) provide indirect evidence both for and against a role for field effects in ictal neural synchronization (Truccolo et al., 2011; Schevon et al., 2012).

Schevon et al., recorded single unit activity during partial seizures with the microelectrode array implanted within the seizure onset zone. In three patients, each of the electrodes detected synchronous unit activity phase locked to the trough of the ictal discharge. However, in two other patients the microelectrode array recorded heterogeneous unit activity (Schevon et al., 2012).

Figure 1A demonstrates marked neural synchrony at the temporal scale of ~10 ms during ictal discharges when the microelectrode array was implanted in the ictal core. To calculate the electric field strength generated by these ictal discharges requires multi-contact depth electrode recordings. However, a rough estimate can be made using prior depth electrode recordings of ictal discharges induced by penicillin application in rabbit cortex (Pockberger et al., 1984). Based on these recordings, the measured ictal discharge amplitude of 1–2 mV in layer 4/5 corresponds with an electric field with a strength of approximately 2–6 mV/mm. Based on *in vitro* (Anastassiou et al., 2011), and *in vivo* (Ozen et al., 2010), evidence this field strength is sufficient to induce population level spike field coherence when the alternating field is applied for an extended duration. Thus, the small variability in the timing of action potentials during ictal discharges suggests that neocortical pyramidal neurons may interact directly via electrical interactions.

**Figure 1:**
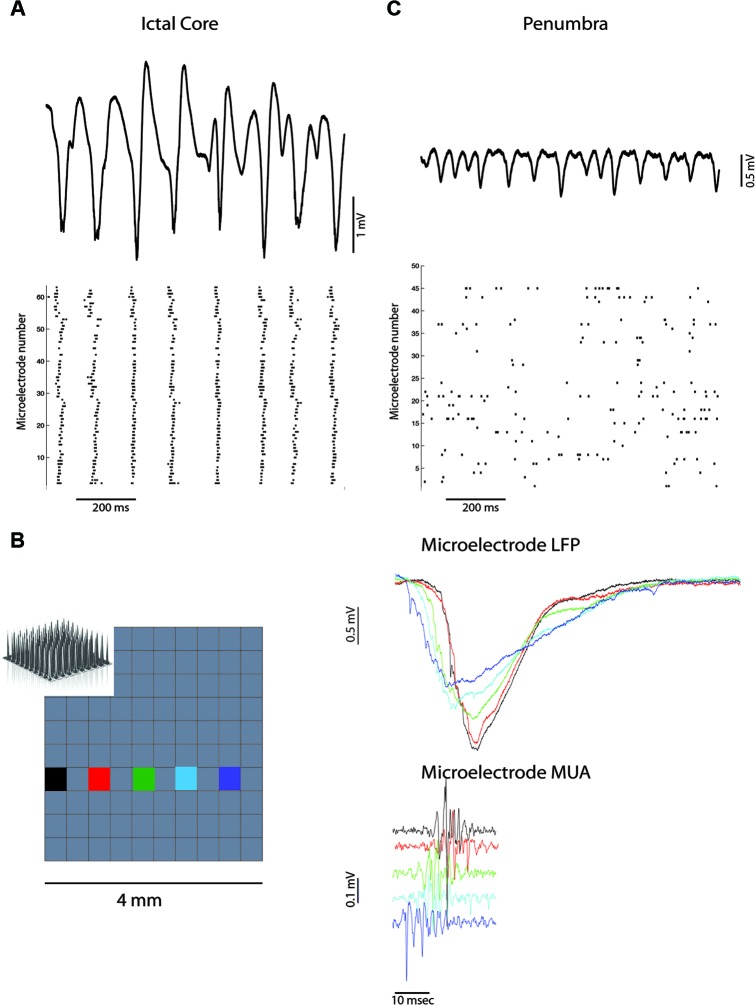
**Ictal discharges are associated with neural synchronization in the ictal core but not the penumbra.** **(A)** Broadband recording of ictal discharges from one of the microelectrodes in the array implanted in the ictal core (above). Corresponding raster plot of multi-unit action potentials recorded from all the active electrodes (below) illustrating synchronization at the scale of 10 ms. **(B)** Propagation of an ictal discharge (right) recorded by the multi-electrode array (left). Corresponding multi-unit activity reflects propagation and the lack of synchronization at the scale of 1 ms. **(C)** Broadband recording of ictal discharges from one of the microelectrodes in the array implanted in the penumbra (above). Corresponding raster plot of multi-unit action potentials illustrate heterogeneity and lack of global synchrony.

Alternatively, neural synchrony during ictal discharges in humans may be solely due to the strong uniform synaptic depolarization and field effects may not play a role. To prove that field effects contribute to neuronal synchronization requires paired intracellular and extracellular recordings from pyramidal neurons during the ictal discharge (Weiss and Faber, 2010). However, paired recordings during ictal discharges recorded from cat neocortex *in vivo* did demonstrate considerable ephaptic depolarization (Grenier et al., 2003a,b).

Thus, neuronal synchrony during ictal discharges may be enhanced in the ictal core by field effect interactions that synergistically pace and entrain the rhythmic paroxysmal depolarizing shifts generated by glutamatergic synaptic transmission (Traub et al., 1985; Parra and Bikson, 2004). Synchronization at the temporal scale of ∼1 ms does not appear to be achieved over extended territories as ictal discharges propagate across the cortex at speeds of ∼500 mm/s (Trevelyan et al., 2007; Schevon et al., 2010, 2012), and action potential firing is affected by the lag times (Figure 1B). This does not rule out the possibility of field effects playing a role in synchronization however, since neocortical slow waves which also propagate rapidly across the cortex (Massimini et al., 2004), can produce fields that enhance and entrain network activity locally (Fröhlich and McCormick, 2010).

 Figure 1C demonstrates the heterogeneous asynchronous firing during the ictal discharges recorded by the microelectrode array in another patient (Schevon et al., 2012). Similar observations of heterogenous asynchronous firing during human seizures have been previously reported (Truccolo et al., 2011). The microelectrode array was implanted in the ictal penumbra in this case. It is apparent that the ictal discharges are smaller amplitude than that recorded from the ictal core in Figure 1A and produce an estimated electric field across the cortical layers of approximately 1–2.1 mV/mm (Pockberger et al., 1984). This field strength should be sufficient to influence spike timing (Francis et al., 2003; Radman et al., 2007a; Anastassiou et al., 2011; Weiss and Faber, 2010), but not necessarily result in strong population level spike field coherence (Ozen et al., 2010; Anastassiou et al., 2011).

Besides a weaker endogenous field, another potential explanation for the heterogeneous, asynchronous firing, in the face of the observed ictal discharges in Figure 1C, is that the neurons in the penumbra have a membrane potential farther from threshold than in the ictal core. Calcium imaging and patch clamp recording from cortical slices bathed in zero magnesium suggest that in the penumbra territory a combination of rhythmic inhibitory post-synaptic potentials (IPSPs) and excitatory post-synaptic potentials (EPSPs) contribute to the ictal discharges (Trevelyan et al., 2006; Trevelyan, 2009; Schevon et al., 2012). Assuming that this is the case in the human ictal penumbra (Figure 1C) the synergistic influence of field effects on neuronal synchronization may be negated. Additional experimental and modeling studies are required to support this hypothesis.

The recordings from the ictal core demonstrate profound neuronal synchrony during ictal discharges. While, paired intra- and extracellular recordings are required to confirm that field effects help to generate this synchronization, the electrical field strength is likely sufficient (Ozen et al., 2010; Anastassiou et al., 2011). In contrast, the recordings from the ictal penumbra highlight how endogenous or exogenous field effects may be affected by synaptic inhibition.

## Conflict of Interest Statement

The authors declare that the research was conducted in the absence of any commercial or financial relationships that could be construed as a potential conflict of interest.

## References

[B1] Anastassiou C. A. Perin R. Markram H. Koch C. (2011). Ephaptic coupling of cortical neurons. Nat. Neurosci. 14, 217–223 10.1038/nn.2727 21240273

[B2] Bikson M. Fox J. E. Jefferys J. G. R. (2003). Neuronal aggregate formation underlies spatiotemporal dynamics of nonsynaptic seizure initiation. J. Neurophysiol. 89, 2330–2333 10.1152/jn.00764.2002 12686586

[B3] Bikson M. Inoue M. Akiyama H. Deans J. K. Fox J. E. Miyakawa H. (2004). Effects of uniform extracellular DC electric fields on excitability in rat hippocampal slices in vitro. J. Physiol. 557, 175–190 10.1113/jphysiol.2003.055772 14978199 PMC1665051

[B4] Deans J. K. Powell A. D. Jefferys J. G. R. (2007). Sensitivity of coherent oscillations in rat hippocampus to AC electric fields. J. Physiol. 583, 555–565 10.1113/jphysiol.2007.137711 17599962 PMC2277040

[B5] Dudek F. E. Yasumura T. Rash J. E. (1998). “Non-synaptic” mechanisms in seizures and epileptogenesis. Cell Biol. Int. 22, 793–805 10873292 10.1006/cbir.1999.0397

[B6] Faber D. S. Korn H. (1989). Electrical field effects: their relevance in central neural networks. Physiol. Rev. 69, 821–863 2546168 10.1152/physrev.1989.69.3.821

[B7] Francis J. T. Gluckman B. J. Schiff S. J. (2003). Sensitivity of neurons to weak electric fields. J. Neurosci. 23, 7255–7261 12917358 10.1523/JNEUROSCI.23-19-07255.2003 PMC6740448

[B8] Fröhlich F. McCormick D. A. (2010). Endogenous electric fields may guide neocortical network activity. Neuron 67, 129–143 10.1016/j.neuron.2010.06.005 20624597 PMC3139922

[B9] Goldensohn E. S. Purpura D. P. (1963). Intracellular potentials of cortical neurons during focal epileptogenic discharges. Science 139, 840–842 10.1126/science.139.3557.840 13948714

[B10] Grenier F. Timofeev I. Steriade M. (2003a). Neocortical very fast oscillations (ripples, 80–200 Hz) during seizures: intracellular correlates. J. Neurophysiol. 89, 841–852 10.1152/jn.00420.2002 12574462

[B11] Grenier F. Timofeev I. Crochet S. Steriade M. (2003b). Spontaneous field potentials influence the activity of neocortical neurons during paroxysmal activities in vivo. Neuroscience 119, 277–291 10.1016/s0306-4522(03)00101-5 12763088

[B12] House P. A. MacDonald J. D. Tresco P. A. Normann R. A. (2006). Acute microelectrode array implantation into human neocortex: preliminary technique and histological considerations. Neurosurg. Focus 20, 1–4 10.3171/foc.2006.20.5.5 16711661

[B13] Jefferys J. G. (1995). Nonsynaptic modulation of neuronal activity in the brain: electric currents and extracellular ions. Physiol. Rev. 75, 689–723 7480159 10.1152/physrev.1995.75.4.689

[B14] Jefferys J. G. Haas H. L. (1982). Synchronized bursting of CA1 hippocampal pyramidal cells in the absence of synaptic transmission. Nature 300, 448–450 10.1038/300448a0 6292731

[B15] Jiruska P. Csicsvari J. Powell A. D. Fox J. E. Chang W.-C. Vreugdenhil M. (2010). High-frequency network activity, global increase in neuronal activity and synchrony expansion precede epileptic seizures in vitro. J. Neurosci. 30, 5690–5701 10.1523/jneurosci.0535-10.2010 20410121 PMC6632330

[B16] Massimini M. Huber R. Ferrarelli F. Hill S. Tononi G. (2004). The sleep slow oscillation as a traveling wave. J. Neurosci. 24, 6862–6870 10.1523/jneurosci.1318-04.2004 15295020 PMC6729597

[B17] Ozen S. Sirota A. Belluscio M. A. Anastassiou C. A. Stark E. Koch C. (2010). Transcranial electric stimulation entrains cortical neuronal populations in rats. J. Neurosci. 30, 11476–11485 10.1523/jneurosci.5252-09.2010 20739569 PMC2937280

[B18] Parra L. C. Bikson M. (2004). Model of the effect of extracellular fields on spike time coherence. Conf. Proc. IEEE Eng. Med. Biol. Soc. 6, 4584–4587 10.1109/IEMBS.2004.1404271 17271327

[B19] Pockberger H. Rappelsberger P. Petsche H. (1984). Penicillin-induced epileptic phenomena in the rabbit’s neocortex I. The development of interictal spikes after epicortical application of penicillin. Brain Res. 309, 247–260 10.1016/0006-8993(84)90591-2 6478219

[B20] Radman T. Datta A. Peterchev A. V. (2007a). In vitro modulation of endogenous rhythms by AC electric fields: Syncing with clinical brain stimulation. J. Physiol. 584, 369–370 10.1113/jphysiol.2007.140947 17702811 PMC2277146

[B21] Radman T. Ramos R. L. Brumberg J. C. Bikson M. (2009). Role of cortical cell type and morphology in subthreshold and suprathreshold uniform electric field stimulation in vitro. Brain Stimul. 2, 215–228, e1–e3 10.1016/j.brs.2009.03.007 20161507 PMC2797131

[B22] Radman T. Su Y. An J. H. Parra L. C. Bikson M. (2007b). Spike timing amplifies the effect of electric fields on neurons: implications for endogenous field effects. J. Neurosci. 27, 3030–3036 10.1523/jneurosci.0095-07.2007 17360926 PMC6672570

[B23] Reato D. Rahman A. Bikson M. Parra L. C. (2010). Low-intensity electrical stimulation affects network dynamics by modulating population rate and spike timing. J. Neurosci. 30, 15067–15079 10.1523/jneurosci.2059-10.2010 21068312 PMC3500391

[B24] Schevon C. A. Goodman R. R. Mckhann G. Jr. Emerson R. G. (2010). Propagation of epileptiform activity on a submillimeter scale. J. Clin. Neurophysiol. 27, 406–411 10.1097/wnp.0b013e3181fdf8a1 21076338 PMC3039548

[B25] Schevon C. A. Weiss S. A. Mckhann G. Jr. Goodman R. R. Yuste R. Emerson R. G. (2012). Evidence of an inhibitory restraint of seizure activity in humans. Nat. Commun. 3:1060 10.1038/ncomms2056 22968706 PMC3658011

[B26] Taylor C. P. Dudek F. E. (1982). Synchronous neural afterdischarges in rat hippocampal slices without active chemical synapses. Science 218, 810–812 10.1126/science.7134978 7134978

[B27] Taylor C. P. Dudek F. E. (1984). Synchronization without active chemical synapses during hippocampal afterdischarges. J. Neurophysiol. 52, 143–155 6086854 10.1152/jn.1984.52.1.143

[B28] Traub R. D. Dudek F. E. Snow R. W. Knowles W. D. (1985). Computer simulations indicate that electrical field effects contribute to the shape of the epileptiform field potential. Neuroscience 15, 947–958 10.1016/0306-4522(85)90245-3 4047402

[B29] Traub R. D. Miles R. Jefferys J. G. (1993). Synaptic and intrinsic conductances shape picrotoxin-induced synchronized after-discharges in the guinea-pig hippocampal slice. J. Physiol. 461, 525–547 10.1016/0304-3940(93)90391-w 8350274 PMC1175271

[B30] Trevelyan A. J. (2009). The direct relationship between inhibitory currents and local field potentials. J. Neurosci. 29, 15299–15307 10.1523/jneurosci.2019-09.2009 19955382 PMC6665954

[B31] Trevelyan A. J. Baldeweg T. van Drongelen W. Yuste R. Whittington M. (2007). The source of afterdischarge activity in neocortical tonic-clonic epilepsy. J. Neurosci. 27, 13513–13519 10.1523/jneurosci.3005-07.2007 18057209 PMC6673106

[B32] Trevelyan A. J. Sussillo D. Watson B. O. Yuste R. (2006). Modular propagation of epileptiform activity: evidence for an inhibitory veto in neocortex. J. Neurosci. 26, 12447–12455 10.1523/jneurosci.2787-06.2006 17135406 PMC6674895

[B33] Truccolo W. Donoghue J. A. Hochberg L. R. Eskandar E. N. Madsen J. R. Anderson W. S. (2011). Single-neuron dynamics in human focal epilepsy. Nat. Neurosci. 14, 635–641 10.1038/nn.2782 21441925 PMC3134302

[B34] Weiss S. A. Faber D. S. (2010). Field effects in the CNS play functional roles. Front. Neural. Circuits 4:15 10.3389/fncir.2010.00015 20508749 PMC2876880

